# Laboratory Diagnosis of Congenital Toxoplasmosis

**DOI:** 10.1128/JCM.00487-16

**Published:** 2016-09-23

**Authors:** Christelle Pomares, Jose G. Montoya

**Affiliations:** a Palo Alto Medical Foundation *Toxoplasma* Serology Laboratory, National Reference Center for the Study and Diagnosis of Toxoplasmosis, Palo Alto, California, USA; b Stanford University, Division of Infectious Diseases, Stanford, California, USA; c INSERM U1065, Centre Méditerranéen de Médecine Moléculaire, Toxines Microbiennes dans la Relation Hôte-Pathogènes, Nice, France; d Service de Parasitologie-Mycologie, Centre Hospitalier Universitaire de Nice, Nice, France; Emory University

## Abstract

Recent studies have demonstrated that screening and treatment for toxoplasmosis during gestation result in a decrease of vertical transmission and clinical sequelae. Early treatment was associated with improved outcomes. Thus, laboratory methods should aim for early identification of infants with congenital toxoplasmosis (CT). Diagnostic approaches should include, at least, detection of Toxoplasma IgG, IgM, and IgA and a comprehensive review of maternal history, including the gestational age at which the mother was infected and treatment. Here, we review laboratory methods for the diagnosis of CT, with emphasis on serological tools. A diagnostic algorithm that takes into account maternal history is presented.

## INTRODUCTION

In 1939, Wolf et al. (1) reported for the first time that the intracellular protozoan parasite Toxoplasma gondii was a pathogen for humans and that it was capable of causing congenital disease as well (1, 2). Following this major discovery, it was promptly recognized that the clinical spectrum of fetuses, newborns, and children congenitally infected with T. gondii could range widely from complete apparent normality to severe neurological and ocular disease and even death (3). It is now well accepted that congenital toxoplasmosis (CT) has a worldwide distribution; recently, the global annual incidence of CT was estimated to be 190,000 cases (95% confidence interval [CI], 179,000 to 206,300), translating into an enormous disease burden of 1.20 million disability-adjusted life years (DALYs) (95% CI, 0.76 to 1.90) per annum (4). However, the morbidity and mortality associated with this disease is preventable, treatable, and reversible (3).

Effective anti-Toxoplasma treatment, when instituted as early as *in utero* during the gestational period, has been shown to significantly decrease mother to child transmission as well as to improve clinical outcomes (5–10). Thus, it is imperative that laboratory tests employed for the diagnosis of CT be sensitive, specific, and exhibit high predictive values in order to promptly identify fetuses and newborns that are likely to significantly benefit from treatment interventions. Here, we review serological tests that are currently available to clinicians for the diagnosis of CT. Additional methods, such PCR, histological stains, isolation of the parasite in mice, and brain imaging studies, and other general laboratory abnormalities associated with the disease are mentioned but are not reviewed in detail since they are beyond the scope of this review. In addition, the relevance of maternal history (e.g., gestational age at which the mother was infected and whether the mother received anti-Toxoplasma treatment or not) is incorporated in a diagnostic algorithm.

## GENERAL CONSIDERATIONS

Congenital toxoplasmosis can occur when a woman acquires T. gondii infection for the first time during pregnancy or, more rarely, shortly before conception. Infection of the fetus occurs when the parasite crosses the hemato-placental barrier and reaches the fetus. The risk of transmission essentially varies with gestational age and whether the mother receives treatment or not (5–10). The overall risk of transmission in mothers who have been treated during gestation is around 30%. However, it varies significantly with the gestational age at which the treated mother acquired the infection, from 15% at 13 weeks, 44% at 26 weeks, and 71% at 36 weeks (10, 11). Less frequently, CT can also occur when women infected in the distant past and prior to gestation reactivate their latent infection due to significant immunosuppression.

The diagnosis of CT can be confirmed or excluded more accurately when comprehensive clinical and laboratory information on the mother and her offspring is readily available to treating physicians. This information significantly affects the interpretation and pretest probability of different laboratory tests (12, 13).

Ideally, it should be established first whether the mother is immunocompromised or immunocompetent and whether she belongs to one of the following three groups: (i) never infected with Toxoplasma and confirmed to remain seronegative 1 month after birth (no risk for CT), (ii) chronically infected—mother acquired her infection prior to gestation (no risk for CT unless she is immunocompromised), or (iii) acutely infected—mother acquired her infection during gestation or within 3 months prior to gestation (at risk for CT). For group 3, it is important to establish (or estimate) the month during gestation at which maternal infection was acquired and whether the mother received anti-Toxoplasma treatment (and if so, which drugs) since the sensitivity and interpretation of laboratory tests can be largely affected by these variables (14). For instance, the sensitivity of serological test results in newborns is lower in those born to mothers who acquired their infection early in gestation and/or received anti-toxoplasmosis treatment during gestation than it is in those born to mothers who acquired their infection late in gestation and/or did not receive treatment (14).

Information on the presence of clinical signs in the fetus and newborn may also be helpful in the interpretation and recommendations, for instance, regarding intervals for follow-up testing after birth or indication for additional tests (e.g., Toxoplasma PCR).

## PRINCIPLES AND METHODS AVAILABLE FOR THE DIAGNOSIS OF CONGENITAL TOXOPLASMOSIS

Several methods have been used for decades for the diagnosis of CT, including detection of Toxoplasma-specific humoral immune responses, amplification of Toxoplasma DNA, identification of Toxoplasma-specific antigen in tissues, and isolation of the parasite (Table 1) (15–17).

**TABLE 1:** Principles and methods used for the diagnosis of congenital toxoplasmosis

Principle	Detection	Platform	Diagnostic of congenital toxoplasmosis
Toxoplasma-specific humoral responses	IgG, IgM, IgA	Dye test, ELISA, and ELISA-like assays, ISAGA, immunofluorescence, agglutination	Positive IgM after 5 days of life and in the absence of blood transfusions. Positive IgA after 10 days of life. Persistence of Toxoplasma IgG beyond 1 year of age
	IgG, IgM, and IgA to specific Toxoplasma antigens	Western blots	Presence of specific bands only seen in the newborn or bands with higher intensity than maternal ones for IgG and/or IgM and/or IgA in a reference laboratory
Toxoplasma nucleic acid amplification	DNA	PCR	Positive result in any body fluid (e.g., amniotic fluid, cerebrospinal fluid^*a*^, peripheral blood, urine)
Immunohistochemistry of Toxoplasma-specific antigens in tissue	Antigens	Immunoperoxidase	Positive result in any tissue (e.g., brain or other fetal tissue)
Visualization by microscopy	Visual identification of tachyzoites and/or cysts	Stains such as hematoxylin/eosin, Giemsa	Positive identification in a reference laboratory
Isolation of Toxoplasma	Whole live parasite	Inoculation in peritoneal cavity of mice	Detection of live cysts from any body fluid or tissue that has been inoculated in mice in a reference laboratory
Brain imaging	Brain calcifications, hydrocephaly, microcephaly	Ultrasound, computed tomography, brain magnetic resonance imaging	Findings can be suggestive but are not diagnostic of CT since other etiologies may result in similar findings
Retinal exam	Inflammation in choroidal and retinal layers	Ophthalmological exam	Retinochoroidal lesions can be highly suggestive or, at times, diagnostic of CT

a In CSF, an extremely high level of protein (e.g., >1,000 mg/dl), presence of eosinophil, and detection of Toxoplasma IgM are also highly suggestive of congenital toxoplasmosis.

### Diagnosis of CT in the fetus.

During gestation, the presence of the parasite in amniotic fluid (DNA amplification, microscopy, or isolation of the organism) and/or fetal tissues (DNA amplification, antigen staining, microscopy, or isolation of the organism) is diagnostic of CT (Table 1). The most commonly used and accepted laboratory method for the diagnosis of CT during gestation is the use of PCR in amniotic fluid, and a positive test result is diagnostic of CT.

### Diagnosis of CT in newborns and infants.

**FIG 1:**
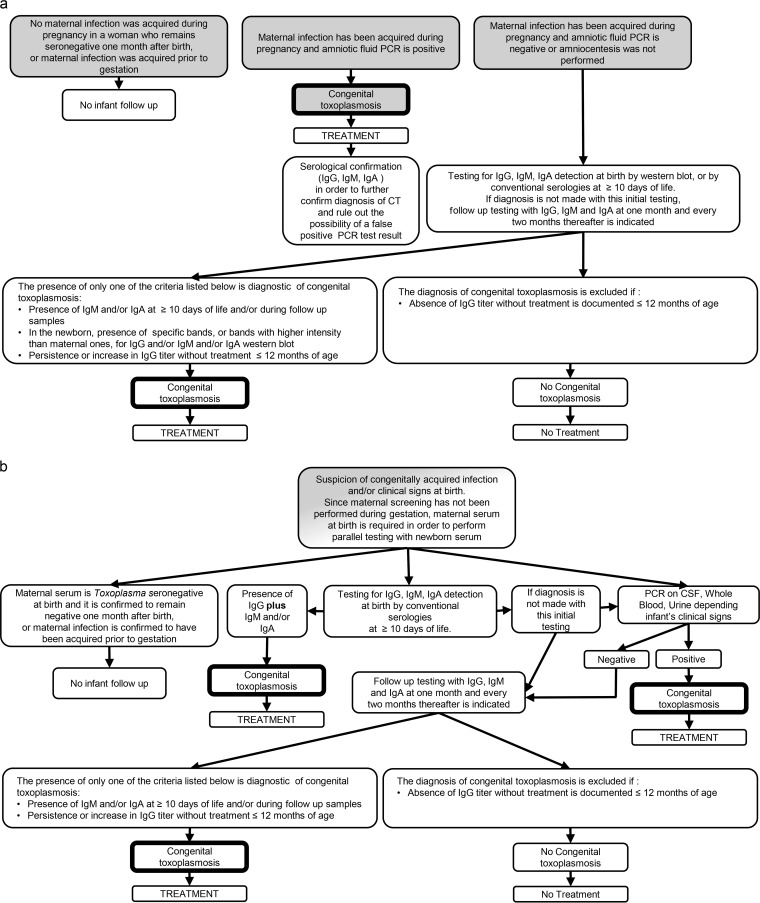
Congenital toxoplasmosis diagnostic algorithm for testing and monitoring infants according to whether maternal antenatal screening and treatment was performed (a) or not (b). Cases in gray and white represent data and/or action before and after birth, respectively.

In the postnatal period, the gold standard to establish a diagnosis of CT is the persistence of Toxoplasma IgG by 12 months of age. Conversely, the standard to rule out the diagnosis is the decrease of Toxoplasma IgG titers until its disappearance at ≤12 months of age in the absence of treatment (Fig. 1).

In the absence of comprehensive clinical history and previous Toxoplasma laboratory test results, diagnosis of CT after the first year of life is confounded by the possibility of the child acquiring infection in the postnatal period. For this reason, all reasonable efforts should be undertaken to diagnose or exclude CT during gestation or the first year of life (infant period).

The most common laboratory method utilized worldwide for the diagnosis of CT in newborns and infants is serological detection of various isotypes of Toxoplasma antibodies in peripheral blood (serum). Although laboratories vary on the choice of specific method to detect Toxoplasma-specific antibodies, Toxoplasma IgG, IgM, and IgA should always be tested since having a combination of IgM and IgA test results, in addition to IgG, has greater sensitivity than either test alone (13, 15–18).

Toxoplasma PCR testing in cerebrospinal fluid (CSF), peripheral blood, and urine can be another laboratory tool that can be used for the early diagnosis of CT and is particularly helpful in regions where antenatal screening and treatment programs have not been implemented (19).

For Toxoplasma IgG detection, the dye test is the reference method and remains the gold standard. However, the dye test can only be performed in reference laboratories due to its dependence on the use of live parasites. Other methods that are more commonly used rely on enzyme-linked immunosorbent assay (ELISA) and ELISA-like assays, agglutination, and indirect immunofluorescence. Many ELISA and ELISA-like assays used for the detection of both Toxoplasma IgG and IgM use automated and closed systems that also allow sequential testing for the detection of IgG and IgM antibodies against other pathogens (17).

For Toxoplasma IgM detection, in addition to the same methods used for Toxoplasma IgG, the IgM immunosorbent agglutination assay (ISAGA) is used and is known for its overall higher sensitivity compared to ELISA and ELISA-like assays (e.g., 81.1% versus 64.8%) (20). The IgM ISAGA is considered to be the method of choice for the detection of Toxoplasma IgM in infants younger than 6 months of age (Table 2).

**TABLE 2:** Overview of sensitivity and specificity of Toxoplasma serological tests in the neonatal period

Principle of the test	Sensitivity (%)	Specificity (%)	Comments	Reference(s)
IgM ELISA and ELISA-like assays	44–81	88.8–100	In two studies, sensitivity was found to be very low (below 30%)	11, 12, 20, 34, 35
IgM ISAGA	44–86.6	77.7–100	IgM ISAGA is the most sensitive test for IgM detection for newborn serology	11, 20, 23, 36
IgA ELISA	52–92.7	64–100	In one study, sensitivity was found to be very low (below 40%)	12, 13, 35–37
IgA ISAGA	52.9–72.5	77.7–97.4		20, 21
IgG Western blotting	33–73.45	96.2–100		25, 26, 38
IgM Western blotting	54.8–78.6	94.74–100		20, 25, 26
Combination of tests				
IgM ISAGA and IgA ELISA	73	98		13
IgG + IgM Western blotting	86.44	94.74		26
IgM + IgA ISAGA and IgG + IgM Western blotting	87.5	81.4		20

For Toxoplasma IgA detection, ELISAs are mostly available. However, IgA ISAGAs are also performed in a few laboratories (20, 21).

Although the use of Toxoplasma IgE was initially reported as promising in some studies, it became subsequently obvious that it did not have any value to the combinatorial power of IgM and IgA (22, 23).

During the postnatal period, the detection of Toxoplasma IgG is confounded by the fact that maternal IgG has been passively transferred across the placenta to the neonate. In addition, the detection of Toxoplasma IgM and IgA in the neonate can also be contaminated with some maternal Toxoplasma IgM during the first 5 days of life and with maternal Toxoplasma IgA during the first 10 days of life. To overcome this challenge, methods to compare maternal and infant IgG, IgM, and IgA profiles have been developed, such as Western blotting. Western blots depict several bands that represent the binding between patient IgG, IgM, or IgA against various known Toxoplasma-specific antigens. The principle is to compare these bands between serum samples from the mother and infant to detect, in cases of congenital toxoplasmosis, autonomous synthesis of antibodies in the infant's serum. This can be established by the presence of bands that are not present in the mother's serum or by bands that have greater intensity in the infant's serum than in the mother's serum. Western blotting has been shown to establish diagnosis up to 3 months earlier than conventional serological methods (24). The sensitivity of Western blotting in combination with conventional serological methods has been shown to be superior to Western blotting or conventional methods used alone. However, the interpretation of Western blots may be difficult and should not be performed after a certain age due to false-positive test results (e.g., for some kits, false-positive results are seen with Toxoplasma IgG and IgM Western blotting after 1 and 3 months of life, respectively) (17, 25, 26).

Most nonreference laboratories can perform the Toxoplasma IgG, IgM, and PCR tests. However, tests such as the IgM ISAGA, Western blotting, and isolation are only performed in reference laboratories and have been validated in infants. In combination with results from conventional tests, they yield a sensitivity that is greater than the sensitivity of each test alone (20, 23).

Few other methods have been described for the diagnosis of congenital toxoplasmosis. The enzyme-linked immunofiltration assay (ELIFA) is an alternative method to compare the immunological profile between mother and infant, but it is performed only in very few laboratories (20). The interferon gamma released after T-cell stimulation by T. gondii antigens has be proven to be useful for the diagnosis of congenital toxoplasmosis, but it is currently not commercially available (27). Detection and follow-up of Toxoplasma IgG in oral fluid have also been used to monitor infants with suspected congenital toxoplasmosis, and it is a promising diagnostic tool (28). In addition, brain imaging studies and retinal exams can also exhibit findings that are highly suggestive of the disease, and in the absence of alternative etiologies and proper clinical context, they can be diagnostic (Table 1).

## OVERALL DIAGNOSTIC APPROACH

Congenital toxoplasmosis can be diagnosed during gestation and/or after birth in the postnatal period. The diagnostic approach to newborns and infants varies significantly depending on whether the mother was screened and treated during gestation and whether a diagnosis of fetal infection was attempted by amniocentesis. Routine prenatal screening and treatment programs have only been implemented in a few countries (Austria, Belgium, France, Norway, Uruguay, and some regions in Italy and Brazil). The clinicians managing newborns born in these regions benefit from having available information, such as maternal serological and amniotic fluid PCR test results, precise gestational age at which the mother was infected, and detailed anti-Toxoplasma treatment history.

In contrast, the vast majority of newborns worldwide, including those in the United States, are born in regions where such programs have not been implemented (23). The absence of or incomplete prenatal screening and treatment has been identified as an important risk factor for congenital toxoplasmosis (29).

## DIFFERENCES IN THE DIAGNOSTIC APPROACH TO CT ACCORDING TO THE PRESENCE OR ABSENCE OF MATERNAL SCREENING AND TREATMENT PROGRAMS

Some important differences are observed in the approach, performance, and utilization of laboratory methods for the diagnoses of CT between regions with Toxoplasma screening and treatment programs during gestation and those without (Fig. 1). The main objective of Toxoplasma maternal screening programs is to diagnose the acute infection during gestation as early as possible. Women who are at risk (Toxoplasma seronegative) are identified during their first prenatal visit and followed at intervals that vary with the program (3). This approach allows for the prompt initiation of treatment of the mothers who seroconvert and of fetuses that become infected. This strategy has been shown to decrease mother to child transmission and severe disease and death in the offspring (9). As a result, fetuses and infants in these regions are diagnosed and treated much earlier than in regions where these programs have not been implemented. An unintended consequence of the screening/treatment approach is that laboratory methods, such as serological and PCR tests, are less sensitive in these regions (12, 23). However, pediatricians and clinicians managing these newborns have immediate access to maternal information and serum, which are critical to the choice of laboratory methods for the diagnosis of CT. For instance, they have available information that is known to influence the choice and performance of laboratory tests, such as precise knowledge about the gestational age at which the mother was infected and whether anti-Toxoplasma treatment was received and, if so, what regimen.

In regions where screening programs are not implemented, critical information on the mother is usually not available. This lack of information often leads to unnecessary laboratory testing in infants who were not at risk of being infected and are later confirmed to be uninfected. In addition, treatment is delayed in newborns that are in fact infected (23). The absence of maternal treatment in regions without screening programs may explain why PCR is more commonly utilized for neonatal diagnosis in these regions since higher sensitivity in PCR assays is expected and has been observed (19, 23).

## SIMILARITIES IN THE DIAGNOSTIC APPROACH TO CT ACCORDING TO THE PRESENCE OR ABSENCE OF MATERNAL SCREENING AND TREATMENT PROGRAMS

The serological approach to the diagnosis of CT in regions with or without these programs is similar once it has been established that the newborn belongs to one of the following three categories (Fig. 1): (i) CT is not likely because mother and newborn are Toxoplasma seronegative, (ii) CT is not likely because the mother is chronically infected (the risk of CT is only present in newborns whose mothers are significantly immunocompromised), or (iii) CT is likely since mother was infected (or is suspected to have been infected) during gestation and/or newborn has clinical signs at birth. Mothers who are Toxoplasma seronegative during gestation and are confirmed to be so 1 month after birth are not at risk for CT. Therefore, serological follow-up in their newborns is not required. The reason to confirm the seronegative status of the mother 1 month after birth is to rule out the rare possibility that the mother may have been infected shortly before delivery.

Mothers who have been confirmed of having acquired the infection in the distant past and prior to pregnancy are not at risk of CT unless the mother is actively immunosuppressed during gestation (e.g., a mother with AIDS who reactivates her Toxoplasma infection or her CD4 count is below 200 cells/mm^3^). Newborns born to these mothers will have detectable Toxoplasma IgG transferred passively from the mother and do not require serological follow-up. However, in regions where maternal screening programs have not been implemented, certainty that the mother has been infected in the distant past and prior to gestation is often impossible. Frequently, the first serum available is obtained in the second trimester or later or at birth. In these situations, when the serological test results exhibit a positive Toxoplasma IgG and IgM, only reference laboratories, such as the Palo Alto Medical Foundation Toxoplasma Serology Laboratory (PAMF-TSL; http://www.pamf.org/Serology/), have serological assays—e.g., IgG dye test, IgM ELISA, Avidity, differential agglutination (acetone [AC]-fixed versus formalin [HS]-fixed tachyzoites) test (AC/HS test), and IgA and IgE ELISAs—that can attempt to establish that the mother was infected in the distant past and prior to gestation (30, 31).

Mothers who have been confirmed of having acquired the infection during pregnancy or shortly before gestation (e.g., within 3 months of conception) are at risk for CT. This risk can be reduced if treatment with spiramycin is initiated. In the United States, spiramycin is available at no cost only by pursuing an investigational new drug (IND) application through the Food and Drug Administration (FDA). In regions where screening programs have been implemented, the diagnosis of maternal infection acquired during gestation is ascertained by seroconversion. In regions without these programs, maternal serum is usually not available or is only available late in gestation or at birth when clinical signs suggestive of congenital infection have been revealed in the fetus, newborn, or infant. With these late serum samples, only testing at reference laboratories, such as PAMF-TSL, has the potential to determine whether mothers were likely to have been infected in the distant past and prior to gestation or during pregnancy. The aim of serological testing in newborns born to these mothers is to confirm, establish, or exclude the diagnosis of CT. In newborns with positive amniotic fluid PCR test results, serological testing and follow-up are still recommended in order to further confirm the diagnosis of CT and to have additional data in cases where the possibility of a false-positive PCR test result is raised. In newborns with negative amniotic fluid PCR test results, or those in whom amniocentesis was not performed, serological testing and follow-up is paramount as a potential diagnostic tool. A newborn serological panel comprised of Toxoplasma IgG, IgM ISAGA, and IgA should be performed regardless of whether the amniotic fluid PCR test was performed and its results. The initial serum should be obtained after 10 days of life in order to avoid misleading results due to potential contamination with maternal blood. Follow-up serum samples should be tested in parallel with the previous sample at 1 month, 2 months, and then every 2 months (Fig. 1). Each newborn born to a mother who has been confirmed of or is suspected to have been infected during gestation or shortly before conception must be followed up serologically until 12 months of age. The Toxoplasma IgG will decrease by half every month until its disappearance around month 6 or 7 in uninfected infants but will not disappear by 12 months of age in infected infants. In some congenitally infected infants, treatment with a pyrimethamine-sulfonamide combination can lead to negativization of the Toxoplasma IgG during follow-up, creating the false sense that the infant is not infected. However, in infected infants, discontinuation of the treatment is followed by a rebound in the Toxoplasma IgG. If IgG remains negative, assuming the infant is capable of producing IgG, the diagnosis of CT is excluded.

## FUTURE DIRECTIONS

The laboratory diagnosis of congenital toxoplasmosis has benefit from various principles and methods (Table 1). Future research should address the cost and feasibility of detection of antibodies, DNA, and live parasite in different platforms and body compartments. For instance, simultaneous detection of multiple analytes in the same assay offers an attractive option for multiplex detection of Toxoplasma IgG, IgM, and IgA and of antibodies against other pathogens with the capacity to cause congenital infection (32, 33). The use of platforms with multiplex capacities can address cost, with the additional benefit that they may be extended to other infections. In addition, the feasibility of testing for antibodies in body compartments beyond serum, such as whole blood and saliva, can address cost and patient convenience (28). Lastly, public health authorities and national policies should address the need to fund and protect reference centers of excellence for the diagnosis and management of congenital infections since these infections may not be seen commonly in individual practices or medical centers but are a source of major morbidity and mortality to the fetus and to newborns.
